# F73. COGNITIVE CLUSTERING IN SCHIZOPHRENIA PATIENTS, THEIR FIRST-DEGREE RELATIVES AND HEALTHY SUBJECTS IS ASSOCIATED WITH ANTERIOR CINGULATE CORTEX VOLUME

**DOI:** 10.1093/schbul/sby017.604

**Published:** 2018-04-01

**Authors:** Kazutaka Ohi, Takamitsu Shimada, Kiyotaka Nemoto, Yuzuru Kataoka, Toshiki Yasuyama, Kohei Kimura, Hiroaki Okubo, Takashi Uehara, Yasuhiro Kawasaki

**Affiliations:** 1 Kanazawa Medical University; 2 Institute of Clinical Medicine, University of Tsukuba

## Abstract

**Background:**

Cognitive impairments are a core feature in schizophrenia patients and are also observed in first-degree relatives of the schizophrenia patients. However, substantial variability in the impairments exists within and among schizophrenia patients, first-degree relatives and healthy controls. A cluster-analytic approach can group individuals based on profiles of traits and create more homogeneous groupings than predefined categories.

**Methods:**

Here, we investigated differences in the Brief Assessment of Cognition in Schizophrenia (BACS) neuropsychological battery (six subscales) among 81 schizophrenia patients, 20 unaffected first-degree relatives and 25 healthy controls. To identify three homogeneous and meaningful cognitive groups regardless of categorical diagnoses (schizophrenia patients, first-degree relatives and healthy controls), cognitive clustering was performed using a k-means clustering analysis approach, and differences in the BACS subscales (verbal memory, digit sequencing, token motor, verbal fluency, symbol coding and Tower of London) among the cognitive cluster groups were investigated. Finally, the effects of diagnosis and cognition on brain volumes were examined.

**Results:**

As expected, there were significant differences in the five BACS subscales among the diagnostic groups (verbal memory, F2,123=20.6, P=1.90 × 10–8; digit sequencing, F2,123=8.0, P=5.65 × 10–4; token motor, F2,123=16.0, P=6.92 × 10–7; verbal fluency, F2,123=14.8, P=1.79 × 10–6 and symbol coding, F2,123=28.8, P=5.64 × 10–11). The cluster-analytic approach generated three meaningful subgroups: (i) neuropsychologically normal (Cluster 1, N=36), (ii) intermediate impaired (Cluster 2, N=60) and (iii) widespread impaired (Cluster 3, N=30). The cognitive subgroups were mainly affected by the clinical diagnosis (χ2=46.7, P=5.33 × 10-10), and significant differences in all BACS subscales among clusters were found (verbal memory, F2,123=64.1, P=8.49 × 10–20; digit sequencing, F2,123=35.7, P=5.89 × 10–13; token motor, F2,123=71.7, P=2.29 × 10–21; verbal fluency, F2,123=84.2, P=9.05 × 10–24; symbol coding, F2,123=115.6, P=5.70 × 10–29 and Tower of London, F2,123=6.9, P=1.43 × 10–3). The effects of the diagnosis (SCZ<FR<HC) and cognitive clusters (Clusters 3<2<1) on brain volumes overlapped in the frontal, temporal and limbic regions. Frontal and temporal volumes were mainly affected by the diagnosis, whereas the anterior cingulate cortex volumes were affected by the additive effects of diagnosis and cognition (FWE-corrected P<0.05, x, y, z=1.5, 40.5, 19.5, T=5.49).

**Discussion:**

We investigated the cognitive heterogeneity and cognitive continuum among schizophrenia patients, first-degree relatives and healthy controls. The cognitive clustering approach without using clinical diagnoses successfully produced more homogeneous cognitive clusters: a neuropsychologically normal, an intermediately impaired and a globally impaired cognitive cluster. Clinical diagnoses (healthy controls, first-degree relatives and schizophrenia patients) were not evenly distributed into the three clusters; i.e., these clusters were mainly affected by clinical diagnoses. Both diagnoses and cognitive clusters were associated with decreased anterior cingulate cortex volumes. Our findings demonstrate a cognitive continuum among schizophrenia patients, first-degree relatives and healthy controls and support the hypothesis that cognitive impairments and the related anterior cingulate cortex volumes would be useful intermediate phenotypes in the pathophysiology of schizophrenia.

